# The role of frontostriatal impairment in freezing of gait in Parkinson's disease

**DOI:** 10.3389/fnsys.2013.00061

**Published:** 2013-10-04

**Authors:** James M. Shine, Ahmed A. Moustafa, Elie Matar, Michael J. Frank, Simon J. G. Lewis

**Affiliations:** ^1^Parkinson's Disease Clinic, Brain and Mind Research Institute, The University of SydneySydney, NSW, Australia; ^2^School of Social Sciences and Psychology, Marcs Institute for Brain and Behaviour, University of Western SydneySydney, NSW, Australia; ^3^Department of Cognitive, Linguistic and Psychological Sciences, Brown Institute for Brain Science, Brown UniversityProvidence, RI, USA

**Keywords:** Parkinson's disease, freezing of gait, functional decoupling, subthalamic nucleus, pedunculopontine tegmental nucleus

## Abstract

Freezing of gait (FOG) is a disabling symptom of advanced Parkinson's disease (PD) that leads to an increased risk of falls and nursing home placement. Interestingly, multiple lines of evidence suggest that the manifestation of FOG is related to specific deficits in cognition, such as set shifting and the ability to process conflict-related signals. These findings are consistent with the specific patterns of abnormal cortical processing seen during functional neuroimaging experiments of FOG, implicating increased neural activation within cortical structures underlying cognition, such as the Cognitive Control Network. In addition, these studies show that freezing episodes are associated with abnormalities in the BOLD response within key structures of the basal ganglia, such as the striatum and the subthalamic nucleus. In this article, we discuss the implications of these findings on current models of freezing behavior and propose an updated model of basal ganglia impairment during FOG episodes that integrates the neural substrates of freezing from the cortex and the basal ganglia to the cognitive dysfunctions inherent in the condition.

## Introduction

Freezing of Gait (FOG) is a common disabling symptom of Parkinson's disease (PD) that typically manifests itself as a sudden inability to walk, despite the intention to move forward (Giladi et al., 1997; Nutt et al., 2011). Alternatively, the condition can also manifest as an inability to turn in a tight circle to traverse through confined spaces, such as narrow doorways (Spildooren et al., 2010). In addition, freezing episodes also occur more frequently during the performance of a concurrent cognitive task while walking (Jacobs et al., 2009; Lewis and Barker, 2009; Spildooren et al., 2010) and the phenomenon has also been associated with deficits in a number of executive functions (Amboni et al., 2008), including impairments in attentional set-shifting (Naismith and Lewis, 2010; Shine et al., 2012a) and response conflict resolution (Vandenbossche et al., 2012; Matar et al., 2013). Due to the vast range of conditions that can either provoke or relieve freezing behavior, there is currently a lack of consensus regarding the underlying mechanism of freezing (Nutt et al., 2011; Shine et al., 2011).

This lack of fundamental understanding of the condition has also severely limited the current therapeutic options for freezing. For example, FOG is known to only show a partial amelioration to dopaminergic medication (Shine et al., 2011) and there is even evidence that some patients only experience freezing during the “on” state (Espay et al., 2012) (though this is a separate syndrome that is not the focus of this paper). In addition, freezing behavior also shows a variable response to deep brain stimulation (DBS) therapy targeting either the subthalamic nucleus (STN) (Niu et al., 2012) or the pedunculopontine nucleus (PPN) (Lewis and Barker, 2009; Follett and Torres-Russotto, 2012). Due to the complex interplay of cognitive, affective, and motor impairments in the disorder, along with variable responses to dopaminergic and electrophysiological therapies, the pathophysiology remains poorly understood at the neural level (Nutt et al., 2011; Shine et al., 2011).

It is clear that a common pathophysiological mechanism of FOG must encompass each of these associated features, linking the factors that *provoke* freezing behavior with those that are involved in the *manifestation* of the symptom. In this perspective, we will use recent insights from neuroimaging experiments to sketch a framework that will link the provocation and manifestation of freezing into a single unified mechanism.

## Evidence from neuroimaging studies

A number of recent studies have used MRI techniques to explore group differences in the structural integrity of the brain. Voxel based morphometry has shown that patients with FOG have impaired gray matter volume in the posterior cingulate cortex and precuneus (Tessitore et al., 2012a). Using resting state functional connectivity, Tessitore and colleagues also reported that patients with FOG have impaired functional connectivity within the frontoparietal networks sub-serving attentional functions (Tessitore et al., 2012b). In addition, studies using diffusion tensor imaging have demonstrated that FOG is associated with poor connectivity between the PPN and the cerebellum (Schweder et al., 2010; Fling et al., 2013), as well as the thalamus and frontal cortex (Fling et al., 2013).

Despite the differences in the structural integrity of the brain in patients with FOG, it is not clear whether the underlying pathophysiology is localized to brainstem or frontostriatal systems. However, the results do broadly suggest impairments in global attentional mechanisms, perhaps implicating thalamic dysfunction (Sadaghiani et al., 2010), or in the effective updating of motor plans, through impaired communication between the cerebellum and the brainstem structures controlling gait (Ito, 2008).

Despite the discovery of these structural abnormalities in patients with FOG, the paroxysmal nature of freezing behavior suggests that the disorder is predominantly functional, manifesting only in response to a specific combination of abnormal neuronal patterns. As such, recent research studies have utilized novel behavioral tasks [such as mental imagery (Snijders et al., 2010) and virtual reality (VR) (Matar et al., 2013; Shine et al., 2013a)] that are able to explore the neuronal abnormalities associated with freezing events in susceptible patients without requiring the execution of actual gait. For example, a recent fMRI study investigated the functional MRI changes associated with a motor imagery task in a group of patients with FOG (Snijders et al., 2010). In this study, patients with and without clinical FOG where required to imagine themselves walking along a presented pathway. Despite the lack of any overt movements in the task, the patients with FOG showed a preferential activation in the mesencephalic locomotor regions (MLR), a brainstem structure associated with the neural control of gait (Lemon, 2008) and the production of anticipatory postural adjustments, which are abnormal in patients with FOG (Jacobs and Horak, 2007; Snijders et al., 2010). As mentioned above, these brainstem regions also show a lack of white matter connectivity to cortical, thalamic, and cerebellar structures (Schweder et al., 2010; Fling et al., 2013). Together, these results suggest that FOG may be due to pathological processes affecting the brainstem structures controlling the processing of gait.

In contrast, functional neuroimaging experiments exploiting VR, where patients use foot pedals to navigate a three-dimensional environment presented on a screen, offer the ability to probe neural activity when a patient is actually performing a motor task. Recent work has demonstrated that freezing behavior can be provoked during this paradigm with patients experiencing paroxysmal episodes where they are unable to move their feet (Naismith and Lewis, 2010). Indeed, the amount of freezing elicited in the VR environment has been correlated with both self-reported (Naismith and Lewis, 2010) and clinically observed FOG (Shine et al., 2012b). The ability to elicit this freezing behavior in the fMRI setting has allowed insights into the neural correlates of FOG (Shine et al., 2013a,b).

One such experiment explored the fMRI differences between periods of normal walking and periods of freezing elicited during the VR task (Shine et al., 2013a). These episodes were associated with a significant increase in the BOLD response within the bilateral dorsolateral prefrontal cortex and the posterior parietal cortices (Shine et al., 2013a), which was consistent with the findings from a number of previous neuroimaging experiments that have implicated frontoparietal dysfunction in the pathophysiology of freezing (Bartels and Leenders, 2008). In addition, the motor arrests were also associated with concomitant decreased BOLD response within the bilateral caudate nucleus, suggesting a potential dissociation between the cortical and subcortical members of the frontostriatal pathways involved in executive function (Alexander and DeLong, 1986). Indeed, a more-recent neuroimaging experiment utilizing the same VR paradigm has shown that motor arrests on the VR task are related to a paroxysmal functional decoupling between the cortical and subcortical regions of the frontostriatal system (Shine et al., 2013c).

Motor arrests on the VR task were also associated with significant abnormalities in the BOLD response within the globus pallidus (GPi), the STN and the MLR (Shine et al., 2013a). Although initially counter-intuitive, one likely explanation put forward for this finding is that during freezing, the GPi and STN enter into an oscillatory state (Timmermann et al., 2007; Spildooren et al., 2010), decreasing their need for oxygen (Buzsáki and Draguhn, 2004) and lowering the relative signal in the BOLD response (Zumer et al., 2010). The oscillatory activity between these two nuclei would ultimately manifest as overwhelming inhibition on the MLR, leading to a decrease in the neuronal firing in this nucleus (Spildooren et al., 2010).

While these conclusions are speculative, there is a wealth of experimental evidence from electrophysiological recording studies that implicate abnormal oscillatory dynamics in the pathophysiology of akinetic Parkinsonian symptoms, particularly in the “theta” (Sarnethein and Jeanmonod, 2007) and “beta” frequency bands (~13–20 Hz) (Hammond et al., 2007; Mallet et al., 2008a,b; Degos et al., 2009; Weinberger and Dostrovsky, 2011). Although there is some consternation about the precise source of these oscillatory signals (McCarthy et al., 2011; Tsang et al., 2012), increased activity in the STN is presumed to play a prominent role (Mallet et al., 2008a,b). Therefore, it is a direct prediction of this theory that freezing episodes should be associated with a temporary increase in beta synchrony within the STN (Brown, 2006), a prediction that is aligned with local field potential recordings in patients with FOG (Weinberger et al., 2006; Singh et al., 2012). In addition, the relative inactivation of the STN following high-frequency DBS surgery has also been shown to alleviate freezing behavior (Niu et al., 2012), however, it is not currently clear whether this is due to inactivation of the STN, decreased oscillatory synchrony between the cortex and basal ganglia or some other related mechanism (Bar-Gad et al., 2003; Hammond et al., 2007; Johnson et al., 2008; Weinberger and Dostrovsky, 2011). These results highlight the key role of subcortical activity in the pathophysiology of FOG, which may reflect an inability to effectively “update” motor sets during ongoing motor task performance (Chee et al., 2009). Furthermore, this interpretation is aligned with recent neurophysiological studies that explored electrical recordings directly from STN and PPN during DBS surgery (Singh et al., 2012; Thevathasan et al., 2012), however, these patterns have not been confirmed during dynamic exploration of specific episodes of FOG.

In addition to these sub-cortical processes, it is clear that there is likely to be a cortical component to the freezing phenomenon. For instance, a further fMRI experiment utilizing the VR gait task highlighted above showed that, although PD patients were able to recruit the dorsolateral prefrontal cortex and the posterior parietal cortex during the dual-processing of concurrent cognitive and motor tasks, those with FOG had decreased activity in the pre-supplementary motor area (pSMA) and the bilateral ventral striatum (Shine et al., 2013b). Although these regions are involved in a number of functions, this limited activity may represent the inability to effectively “shift” between competing attentional networks (Spildooren et al., 2010; Menon, 2011; Nutt et al., 2011; Shine et al., 2011) or could indicate an impairment in processing of error-related neuronal signals during the VR task (Salamone and Correa, 2002). This failure in error processing could relate more specifically to dysfunction within the hyper-direct pathway of the basal ganglia, which links specific regions of the frontal cortex (including the pSMA) with the STN of the basal ganglia (Nachev et al., 2008; Cavanagh and Frank, 2013; Haynes and Haber, 2013) and has previously been suggested to have a role in the processing of response conflict (Cavanagh et al., 2012). Furthermore, a role for the hyper-direct pathway in FOG is aligned with a recent electroencephalography (EEG) study that has shown that the transition from walking to freezing is associated with large increases in activity within the theta frequency sub-band (Handojoseno et al., 2012). This shift in EEG frequency has been broadly aligned with the response to conflict processing in the motor and pre-motor cortices (Nachev et al., 2008) and has also been related to increased response caution, reflected in a slowing of reaction time during conflict (Cavanagh et al., 2011). Furthermore, conflict-related theta power in cortical scalp electrodes was mirrored in the activation of STN local field potentials, further implicating the hyper-direct pathway in conflict processing (Cavanagh et al., 2011).

Together, these studies highlight the role of dysfunctional neuronal processing at multiple hierarchical levels of the nervous system, including brainstem and spinal cord structures that control gait and posture, frontoparietal cortical regions that subserve executive functions with subcortical basal ganglia structures link the two levels together.

## An updated model of freezing behavior

A key feature of freezing behavior is that it can be triggered by a range of differing factors including the processing of cognitive (Lewis and Barker, 2009), sensorimotor (Almeida and Lebold, 2010; Ehgoetz Martens et al., 2013) and limbic/affective information (Ehgoetz Martens et al., 2013). Although on the surface these aspects of brain function all appear quite dissimilar, they can all potentially lead to a state of increased response conflict processing in the brain in patients with PD (Vandenbossche et al., 2012).

**Figure 1:**
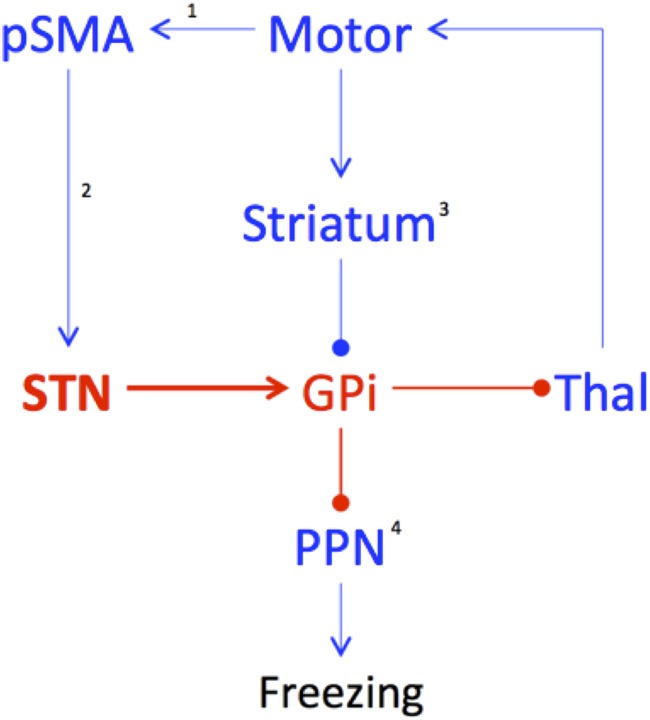
**Freezing is due to decreased input and output of the basal ganglia.** In periods of high response conflict, the subthalamic nucleus (STN) increases its firing rate, which subsequently drives increased activity within the internal segment of the globus pallidus (GPi), leading to decreased activity within the thalamus (Thal) and the pedunculopontine nucleus (PPN). While the triggering event in this model is currently unknown, it may be due to: ^1^—inherent impairment within the pre-supplementary motor area (pSMA), leading to an inefficient communication with the STN; ^2^—impairments in the temporal dynamics between the pSMA and STN, possibly due to impaired white matter connectivity; ^3^—cellular deficits within the striatum that place the nucleus at an increased risk of becoming hyperpolarized; or ^4^—pathology within the PPN, priming the nucleus for hyperpolarization by minimal inhibitory input. Freezing may be due to any combination of these factors. Key: red—increased activity; blue—decreased activity; arrow—excitatory connection; circle—inhibitory connection.

Through its connection to the pSMA (the so-called “hyper-direct” pathway of the basal ganglia), the STN is able to bypass the striatum and directly drive an increase in inhibitory GABAergic output from the output structures of the basal ganglia, such as the internal segment of the GPi. Increased activity in the GPi, which is a member of the direct pathway of the basal ganglia, leads to an increase in the rate of inhibitory output onto the brainstem and thalamic structures that control the output of effective motor behaviors (Frank, 2006) (see Figure 1). Given its key role in regulating activity in the basal ganglia circuitry, the STN is therefore, able to modulate response conflict until such time as an appropriate response can be made (Frank, 2006). Through its processing of conflict-related neuronal signals and the modulation of inhibitory tone within the basal ganglia, the STN therefore, serves as a putative neuroanatomical link between both the provocation and manifestation of freezing behavior. During the processing of increased conflict, regardless of its modality (e.g., processing cognitive information whilst maintaining motor output), activity within the STN could act to “trigger” overwhelming increases in the output structures of the basal ganglia, effectively shutting down the targets through GABAergic inhibition.

Given the specific patterns of connectivity within the basal ganglia circuitry, the likely sequelae of impaired striatal activity is that the output structures (the GPi internus and the substantia nigra pars reticularis) will enter into low-energy oscillatory states, coupling with structures such as the STN (Buzsáki and Draguhn, 2004; Frank, 2006; Spildooren et al., 2010). Indeed, recent mechanisms of basal ganglia function have proposed that the primary role of the basal ganglia network is in the modulation of emergent synchronous oscillatory patterns rather than the alteration of single synaptic patterns (Bar-Gad et al., 2003; Hammond et al., 2007; Weinberger and Dostrovsky, 2011). The alternation between relative excitation and inhibition in the basal ganglia (Chee et al., 2009) would therefore, manifest as an inability to effectively “select” a single motor plan, which would ultimately lead to the overwhelming inhibition of the efferent targets of the output nuclei (such as the relay nuclei of the thalamus) and the brainstem structures controlling gait, such as the PPN (Tsang et al., 2010) and other members of the MLR (Snijders et al., 2010). Decreased activity within the dorsal PPN would impair motor initiation, due to the nuclei's efferent connectivity with the central pattern generators in the spinal cord that control coordinated flexion and extension of the muscles controlling gait (Takakusaki et al., 2004).

These proposed roles of the STN are well supported by behavioral (Aron and Poldrack, 2006; Frank, 2006; Wiecki and Frank, 2010) and neuroimaging evidence (Aron et al., 2007; Haynes and Haber, 2013). For example, the STN has a well known association with set-shifting behavior (Tsang et al., 2012), which is impaired in patients with freezing (Shine et al., 2012a). Furthermore, the role of the STN in freezing is aligned with the functional neuroimaging studies that suggested that the GPi and the STN entering into a low energy oscillatory state during freezing behavior (Buzsáki and Draguhn, 2004; Spildooren et al., 2010). In the low dopaminergic state, this oscillatory activity would then strongly facilitate increased activity within both the direct and indirect pathways of the basal ganglia, leading to overwhelming inhibition on the output structures of the basal ganglia, such as the thalamus and the MLR, but also on the striatum itself, leading to a functional decoupling between the cortex and the striatum. This is precisely the pattern of BOLD response changes that were observed during motor arrests on the VR task (Snijders et al., 2010) and have recently been reproduced during freezing episodes in an upper-limb tapping task (Vercruysse et al., 2013). Finally, the model also receives supportive evidence from a study of PD patients, in which high-frequency STN DBS led to a decrease in the normally elongated reaction times during conflict processing (Frank et al., 2007).

**Figure 2:**
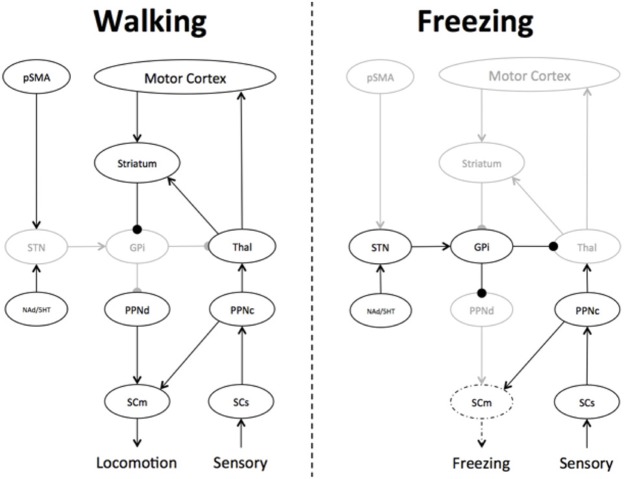
**Differences in basal ganglia connectivity during Walking and Freezing.** During walking, the Pre-supplementary Motor Area (pSMA) and Motor Cortex are able to effectively communicate motor plans to the basal ganglia, leading to the effective gating of basal ganglia outflow, allowing the appropriate communication of motor plans to the brainstem structures controlling gait, such as the dorsal pedunculopontine nucleus (PPNd), which subsequently informs the motor spinal cord (SCm), leading to normal gait. In addition, there is also effective feedback from the sensory spinal cord (SCs) to the cholinergic PPN (PPNc), further informing gait and balance. During Freezing, overwhelming response conflict leads to an increase in the firing rate within the subthalamic nucleus (STN), which then drives an increase in activity within the globus pallidus internus (GPi), effectively decreasing the output of the basal ganglia, respectively. The inhibited PPNd is then unable to communicate effective motor plans to the SCm, however, the SCs is able to remain in communication with the PPNc, leading to an imbalance in activity within the greater PPN and abnormal activation patterns in the SCm. The overwhelming inhibitory state of the basal ganglia can only be broken by a focused, goal-directed action, which would trigger the striatum to inhibit the GPi, effectively releasing the STN-mediated “brake” on the PPNd and the SCm. Key: black—active; gray—hypo-active; dotted line—mixed active/hypoactive.

The oscillating inhibitory state of the basal ganglia nuclei may also explain the poorly understood phenomenon of “trembling in place,” which refers to lower limb oscillatory activity in the 5–7 Hz range commonly observed during episodes of FOG (Moore et al., 2012). Computational modeling experiments have shown that Parkinsonian tremor (which occurs in the same 5–7 Hz frequency band) emerges naturally due to rhythmic activity between the STN and the GPi externus in the presence of a dopaminergically-depleted basal ganglia (Pahapill and Lozano, 2000). As such, the inhibitory output from the basal ganglia would therefore, oscillate at the same frequency, rhythmically inhibiting and relaxing its inhibition on the PPN. The moments of relative quiescence on the PPN would therefore, allow increased signaling from the cholinergic cells within the PPN, which would still remain active via its connections with sensory signals via the dorsal root of the spinal cord (Pahapill and Lozano, 2000) (see Figure 2). This mechanism also provides a potential explanation for the relationship between the freezing phenomenon and impaired postural responses during tests of balance function (Jacobs and Horak, 2007).

## Insights into potential pathological processes

One of the major implications of this model is that freezing is best conceptualized as a functional disorder that only manifests once certain circumstances have occurred. This raises an interesting question regarding the likely location of pathology in the brains of patients with PD and freezing behavior. Based on the model, any pathological process that impaired the capacity of the brain to deal with information processing, and thus, to manifest increased conflict signaling would lead to an increase in freezing behavior.

Although there are many regions of the brain in which pathology would lead to increased global conflict, the most likely candidates are the ascending neurotransmitter projection systems of the brainstem, such as the ventral tegmental area, the locus coeruleus, and or the dorsal raphe nucleus. Each of these nuclei sends modulatory neurotransmitters to large portions of the brain, including both cortical and subcortical sites involved in walking and executive function. Indeed, these regions are often the target of Lewy body pathology (Rye and DeLong, 2003) and some of the projection nuclei, such as the dorsal raphe (Xiang et al., 2005) and the ventral tegmental area (Oades and Halliday, 1987), have direct efferent connections with the basal ganglia. Although each of the neurotransmitter systems has different effects on target neurons, imbalances in the proportion and timing of these neurochemicals is likely to lead to inefficient neuronal processing on a global scale, priming the system for errors and hence, increased response conflict.

Another possible candidate region is the PPN (Mazzone et al., 2013), which has also been associated with Lewy body pathology in PD (Pahapill and Lozano, 2000). The PPN has extensive efferent connections with the STN and also with a number of other structures that modulate the brain's response to conflict, such as the intralaminar nuclei of the thalamus and the striatum (Pahapill and Lozano, 2000). In rats, low-frequency stimulation of the PPN leads to decreased firing in the STN (Alam et al., 2012) and as such, impairments in the effectiveness of this signaling pathway could potentially lead to an increase in the influence of the STN over the basal ganglia. These concepts are supported by the functional improvements experienced by patients with freezing following DBS of the PPN nucleus (Stefani et al., 2007; Strafella et al., 2008; Ferraye et al., 2010; Hamani et al., 2011; Tykocki et al., 2011; Follett and Torres-Russotto, 2012; Khan et al., 2012), along with increases in regional cerebral blood flow observed during stimulation of the PPN (Strafella et al., 2008; Ballanger et al., 2009). In addition, this interpretation is consistent with the finding that the PPN lacks appropriate white matter connectivity with the cerebellum in patients with FOG and PD (Tessitore et al., 2012b; Fling et al., 2013), however, this finding could also be explained as the by-product of decreased firing within the PPN as a result of overwhelming inhibition from the basal ganglia (Lewis and Barker, 2009), suggesting that the primary pathology in freezing may not reside in the PPN.

It is also possible that the proposed increase in STN oscillatory activity is due to a dysfunctional process within the pSMA (see Figure 1). For example, global states that predispose the brain to impaired information processing may preferentially affect the pSMA, particularly in its ability to effectively select motor plans to match both exogenous affordance patterns and internal motivational states (Nachev et al., 2008). Due to an inability of the pSMA to switch between competing motor plans, the STN may receive inappropriate information from the pSMA—either of multiple, competing motor plans (Nachev et al., 2008) or the correct plan on a time-delay (Hikosaka and Isoda, 2010). A failure of information processing would cause the STN to increase its firing rate, leading to inhibition of the input and output structures of the basal ganglia, thus, effectively “buying time” until the appropriate, goal-directed behavior can be transmitted by the cortex (Frank, 2006; Spildooren et al., 2010).

## Future directions

Although the separate predictions of these different hypotheses may be difficult to dissociate with fMRI, measures with higher temporal resolution may help to clarify the precise role of each structure in the spatiotemporal evolution of a freezing episode. As such, future studies should now be constructed to test the different aspects of this model using an array of neuroscientific techniques. Firstly, activity within the STN and PPN could potentially be recorded directly during DBS surgery, allowing for the analysis of the time course of activation and deactivation patterns within the different nuclei with respect to freezing behavior. Future neuroimaging experiments should explore the presence or absence of impairments in functional and effective connectivity associated with the predictions of the model, with a particular emphasis on the dynamic connectivity between cortical and subcortical structures. Finally, computational modeling experiments could be designed in order to probe the dynamic elements of the model, focusing on whether abnormal conflict processing through the STN can predict the specific behavioral patterns displayed on different neuropsychological and motor-based tasks by patients with freezing. Together, the results of these studies will help to inform the next generation of therapeutic advances for freezing behavior in PD, including the utilization of targeted closed-loop DBS (Rosin et al., 2011; Tsang et al., 2012) and the discovery of novel locations for DBS electrode placement (Stefani et al., 2007; Lourens et al., 2011; Khan et al., 2012).

### Conflict of interest statement

The authors declare that the research was conducted in the absence of any commercial or financial relationships that could be construed as a potential conflict of interest.
